# The influence of infant sleep problems and sleep training on maternal subjective well-being

**DOI:** 10.4102/hsag.v29i0.2660

**Published:** 2024-07-05

**Authors:** Jacomien Muller, Tharina Guse

**Affiliations:** 1 Department of Psychology, Faculty of Humanities, University of Pretoria, Pretoria, South Africa

**Keywords:** infant and child sleep problems, behavioural sleep intervention, sleep training, subjective well-being, perceived stress, mothers, Southern Africa, quantitative

## Abstract

**Background:**

Disturbed sleep during infancy and early childhood can have a detrimental effect on parental sleep and consequently parental well-being. However, there is a paucity of research on how perceived child sleep problems and behavioural sleep interventions as treatment influence maternal subjective well-being.

**Aim:**

This study aimed to explore the incidence of subjective well-being in mothers of children with sleep problems and whether implementing two behavioural sleep interventions changed their well-being.

**Setting:**

The research was conducted in a community setting, with parents voluntarily approaching a sleep consultancy based in South Africa.

**Method:**

Using data from 119 mothers voluntarily approaching a sleep consultancy in South Africa, a pre-test-post-test design was employed to investigate changes in life satisfaction, affect, couple satisfaction, perceived stress and depression approximately 3 weeks after implementation of a sleep intervention.

**Results:**

Results indicate that mothers of children with sleep problems experienced moderate to high life satisfaction and positive affect although the presence of moderate negative affect, couple satisfaction and stress and mild depression suggest possible decreased subjective well-being.

**Conclusion:**

Findings suggest implementation of graduated extinction and extinction with parental presence sleep interventions may improve life satisfaction, affect, stress and depression but not couple satisfaction in the short-term.

**Contribution:**

This study contributes towards understanding the effect of sleep loss on the subjective well-being of mothers and provides preliminary evidence regarding the benefits of two sleep interventions for improving maternal subjective well-being.

## Introduction

While good quality sleep is an important aspect of daily functioning (Davidson et al. 2019), child sleep patterns differ from those of adults and sleep problems are highly prevalent during early childhood (Williamson et al. 2020). Disturbed sleep during infancy and early childhood can negatively impact parental sleep. Because it is advised that adults aim for 7 h of high-quality, well-timed and regular sleep each night, free from interruptions or sleep disturbances (Watson et al. 2015), child sleep problems may deleteriously influence parental well-being.

Parents may elect to implement behavioural sleep interventions (BSI) (colloquially termed *sleep training*) to improve perceived child sleep problems during infancy and early childhood. Extant literature suggests that poor child sleep deleteriously influences parental well-being (Hall et al. 2017). Behavioural sleep interventions are both efficacious for child sleep and may improve the well-being of parents after implementation (Meltzer & Mindell 2014). However, there is a dearth of research on BSI in African countries. Little is known about how parental subjective well-being (SWB) (specifically, life satisfaction and positive and negative affect) is influenced by perceived problematic child sleep or how implementing BSI (whether successful or not) influences SWB. Subjective well-being is associated with both individual and family functioning, and improved health-related outcomes and relationships (Diener, Lucas & Oishi 2018). Any impact of BSI implementation on the SWB of parents should therefore be considered, as changes in the well-being of parents may influence the family system (Newland 2015), and the well-being of some or all members of the family. As such, we aimed to explore the SWB of parents reporting child sleep problems, and whether SWB changed after BSI implementation, at a sleep consultancy based in South Africa.

## Subjective well-being

In our study, we conceptualised well-being from the hedonic perspective, which equates well-being to the presence of positive affect and life satisfaction, and the absence of negative affect. Subjective well-being relates to how subjectively satisfied someone is with their life (Diener et al. 2018). Diener (1984) proposed a tripartite model of SWB, consisting of an assessment of one’s life satisfaction, positive affect and negative affect. Evaluations of SWB therefore include both cognitive and affective responses to life situations (Diener et al. 2017).

Judgements on life satisfaction may relate either to one’s life, broadly construed or individual aspects of one’s life, such as marriage (Diener et al. 2018). A high incidence of positive affect and low incidence of negative affect indicates the presence of SWB, whereas several factors contribute to a decrease or absence of well-being, including changes in circumstances such as marriage (Diener et al. 2017) and perceived stress (Atanes et al. 2015). Respondents determine the factors that contribute to their life satisfaction and what constitutes the good life. When parents seek treatment for perceived child sleep problems, it therefore suggests that they are unsatisfied with this domain of their life. We included measures of perceived stress and depression to determine the absence of well-being and confirm prior findings in a Southern African setting, which has not been explored in this context before.

### Defining child sleep problems

Because of differences in sleep patterns between adults and infants, adult sleep can be disrupted, particularly in families where children present with sleep difficulties. While parameters for child sleep problems have been suggested (see Paavonen et al. 2020), most researchers employ parent reports to characterise sleeping difficulties (Field 2017; Mindell et al. 2006). Consequently, definitions may vary greatly and are subject to various influences, such as culture and knowledge of child sleep requirements. In this regard, a sleep onset latency of 30 min may concern one parent, whereas another will not be concerned if their child presents with a sleep onset latency of 45 min. Child sleep problems are therefore often defined as parentally described extended sleep onset latency, repeated night-waking and brief total night-time sleep (Field 2017). In this regard, the International Classification of Sleep Disorders specify that child sleep is problematic if parents perceive it as such (Whittall et al. 2021). Perceived child sleep problems might thus drive parents to seek treatment.

### Parental well-being and child sleep problems

Prior research has confirmed an association between decreased parental mood and sleep deprivation resulting from poor child sleep, as evidenced by higher scores of parental depression (Hall et al. 2017). In addition, higher parental stress (Hall et al. 2017) and family stress and tension (Hiscock & Fisher 2015) are associated with exhaustion and disrupted sleep, thus affecting parental well-being broadly.

However, there is a dearth of literature regarding how problematic child sleep may specifically impact parental SWB. Satisfaction with life may be deleteriously impacted by poor child sleep as decreased mood, increased stress and insufficient sleep in adults associated with decreased life satisfaction (Zhi et al. 2016). Affect may be impacted in that sleep loss, regardless of problematic child sleep, is associated with increased negative affect, such as anger, and less positive affect, such as joy (Gordon & Chen 2014). Research further suggests that mothers report elevated irritability because of fragmented sleep (Hiscock & Fisher 2015). Because increased positive affect and decreased negative affect are central measures of SWB, changes in affect on account of sleep loss may impinge on parental SWB.

As parental SWB may be adversely influenced by disturbed child sleep, it is important to explore approaches to minimise potential deleterious consequences. Behavioural sleep interventions, concerned with changing and enhancing problematic child sleep, is an approach that may be investigated.

### Behavioural interventions for treatment of child sleep problems

Previous studies suggest that BSI reduces child sleep problems, with concomitant improvements in parental sleep and well-being (Field 2017; Honaker et al. 2018; Symon & Crichton 2017).

Several behavioural interventions have been designed to address problematic child sleep, principally aimed at improving independent child sleep onset and maintenance and reducing negative sleep associations (Honaker et al. 2021). Prior studies have established the effectiveness of two intervention programmes (graduated extinction [GE] and extinction with parental presence [EwPP]) for settling problems as well as frequent night-wakings (Meltzer & Mindell 2014). Established beneficial outcomes for parental well-being include improved mood (Hall et al. 2017) and decreased stress (Field 2017). However, extant literature on couple satisfaction is contradictory, with both improvement (Meltzer & Mindell 2014), and no improvement (Smart & Hiscock 2007) reported.

To date, there are limited studies that have explored the impact of BSI on satisfaction with life and affect on parents. While there is a paucity of BSI literature focussed on parental well-being in terms of negative affect, it appears that anger and frustration improve after the implementation of BSI (Hall et al. 2017; Symon & Crichton 2017), likely because parental involvement and responsibilities around child sleep decline. Only one study considered the association between BSI and positive affect (pleasure), finding that pleasure improved profoundly for parents after a sleep intervention (Symon & Crichton 2017).

While the efficacy of BSI has been established, the influence of its implementation, whether successful or not, on affect (either positive or negative) has rarely been considered, and the influence on life satisfaction has not been explored. Additionally, sleep problems are reported relatively similarly across different studies and cultures (Reuter et al. 2020), but there is a lack of research on BSI in the African context. This study addresses the gap in the research, exploring two BSI techniques (GE and EwPP) in a Southern African population. Specific domains of well-being measured in this study include life satisfaction, positive and negative affect, couple satisfaction (as a domain of life satisfaction), postnatal depression and stress (as indicators of the absence of SWB).

## Research methods and design

### Study design

We utilised a pre-test-post-test design within a community setting. Parents who voluntarily approached a South African sleep consultancy for assistance were recruited to participate in the study. In order to prevent selection bias, the research information, together with the electronic survey, was sent to all clients approaching the sleep consultancy. Willing participants electronically returned the surveys. Pre-test measures were completed within 2–3 days prior to BSI implementation to establish a baseline. The duration of the intervention was between 2 to 3 weeks, with follow-up post-test measures completed approximately 3 weeks afterwards. This timeframe allowed 3 to 4 weeks from the end of the intervention for parental sleep to normalise, should the intervention be successful. Efficacy of sleep interventions is high, with rates over 80% (Honaker et al. 2018). Given the high likelihood of a successful intervention, enough time was therefore provided that parental sleep could normalise prior to the post-test. Because of the context of the study (a community setting where participants voluntarily approached a sleep consultancy and were then immediately assigned to a sleep consultant within their area), a control or waitlist group was not possible.

### Behavioural sleep intervention procedure

The participants followed a support-intensive BSI programme in this study. Each family was assigned a sleep consultant, who conducted an intake survey with the parents to acquire detail regarding current sleeping behaviour. Sleep consultants then decided on the intervention based on child age (EwPP for children under 1 year of age; GE for children over 1 year) and parental preference (EwPP for parents who were intolerant to crying for children over 1 year). During a virtual consultation lasting approximately 2 h, the sleep consultants educated parents on matters related to child sleep and explained the intervention plan (either GE or EwPP). The education addressed issues such as diet, screen time, positive bedtime routines, daytime naps, age-appropriate child sleep requirements and an appropriate sleep environment.

In a GE intervention, parents were told to put the child to bed following a positive bedtime routine, then exit the room for a specific duration (based on the child’s age and parental choice). If the child became agitated within the allocated time, parents could return and interact with the child for a few moments. This could take the form of verbal or physical interaction and could be escalated if the child did not settle. In an EwPP intervention, parents systematically decreased their presence in the bedroom throughout a set number of days, always starting with a positive bedtime routine.

Parents offered verbal or physical comforting if a child called and increased the level of support if the child required it. During the intervention period, which typically lasted 2 weeks, the assigned consultants were on hand remotely to offer support and advice should the families require it. Daily calls were made to the parents to follow up on the previous night, and families were asked if they could maintain sleep logs for the consultants to track progress. It should be noted that the interventions took place in a community setting and were thus outside of the control of the researchers. Therefore, when parents requested it from the sleep consultants during the intervention, they were moved to a different intervention type (either EwPP or GE, depending on their preference).

### Participants

Parents of children (up to age five) with perceived problematic sleep, who willingly sought out a South African sleep consultancy, and who were within committed relationships (as a subdomain of life satisfaction), were recruited to participate in the study. Only mothers opted to participate in the study. *A priori* power analysis using G*Power version 3.1.9.7 (Faul et al. 2009) estimated the required sample size to be approximately 34 in each group at pre-test and post-test level to achieve an 80% power for detecting a medium effect, at a significance criterion of α = 0.05. At baseline, 119 mothers elected to participate (*M*_*Age*_ = 32.83, *SD* = 4.09). The majority of the mothers were from South Africa (84%), followed by Zimbabwe (9.2%), and most (66.4%) had one child in the household. Children’s ages in the sleep intervention at baseline were from 3 to 35 months (*M* = 9.34, *SD* = 5.70).

At follow-up, only 77 mothers completed the measures (*M*_*Age*_ = 33.03, *SD* = 3.92), with similar demographics as at baseline. The majority of the mothers in the follow-up were from South Africa (84.4%), followed by Zimbabwe (11.7%). Most participants (61%) had one child in the home, and the children’s ages ranged between 3 and 24 months (*M* = 9.38, *SD* = 5.24). Most mothers were employed (84.4%). At follow-up, the attrition rate was 35%, thus increasing the risk of attrition bias. While high, this dropout rate is similar to prior research (Whittall et al. 2021). It should also be noted that data were collected over the period 01 March 2020 to 10 February 2021, and the coronavirus disease 2019 (COVID-19) pandemic severely impacted response rates at follow-up. Reasons for not responding during follow-up included struggles in accessing necessary technology (computers and printers), challenges in daily life during lockdown, lack of time and work and family commitments.

### Measures

#### The Satisfaction with Life Scale

The Satisfaction with Life Scale (SWLS) is designed to measure life satisfaction as a whole (Diener et al. 1985). It consists of only 5 items and have been utilised to assess the life satisfaction aspect of SWB in many different contexts, with good psychometric properties, including internal and test-retest reliability (Pavot & Diener 2008). Items are answered on a 7-point Likert scale with total scores ranging between 5 and 35. Low scores indicate less satisfaction, whereas high scores indicate high satisfaction with life. Midlevel scores suggest individuals are generally satisfied with life, with aspects that could be enhanced (Pavot & Diener 2008). Previous research established a mean Cronbach alpha coefficient of 0.78 (Corrigan et al. 2013), while a Cronbach alpha coefficient of 0.85 was achieved in the current study.

#### The Scale of Positive and Negative Experience

The Scale of Positive and Negative Experience (SPANE) evaluates how often an individual has felt positive and negative emotions in the last 4 weeks (Diener et al. 2010). It is used to assess the affective component of SWB, with good validity and reliability (Du Plessis & Guse 2017). It consists of two subscales: SPANE N for negative affect and SPANE P for positive affect, each subscale containing six items. Participants must rate their emotional experiences on a scale of 1–5. The subscale scores range from 6 to 30, with higher scores indicating higher positive or negative emotions, and a score of 12 indicating the midrange. The SPANE consists of general negative and positive feelings, such as *good* or *bad*, and distinct negative and positive emotions such as *afraid* and *happy* (Diener et al. 2010). Previous research has established Cronbach alpha coefficients between 0.81 to 0.90 (Diener et al. 2010), while a Cronbach alpha coefficient of 0.93 for the SPANE-P and 0.87 for the SPANE-N was achieved in the current study.

#### The Couples Satisfaction Index

The Couples Satisfaction Index (CSI)-16 is a concise tool for evaluating relationship satisfaction in couples presently in a relationship, with good reliability and validity (Quinn-Nilas 2023), and can be used for different types of romantic relationships (e.g., living together or married) (Funk & Rogge 2007). The scale is comprised of different response types with some items arranged over six (nine items) and seven (one item) point Likert scales, and the relationship is described on a six-point bipolar adjective scale (six items). The scores are added, with a range of 0–81, where higher scores indicate greater satisfaction with the relationship, while those falling under 51.5 indicate dissatisfaction in a relationship (Funk & Rogge 2007). Previous research established Cronbach alpha coefficients of 0.98, while a Cronbach alpha coefficient of 0.96 was found in the present study.

#### The Perceived Stress Scale

The Perceived Stress Scale (PSS)-10 is designed to assess an individual’s perception of life and specific circumstances as uncertain, overwhelming and stressful (Cohen, Kamarch & Mermelstein 1983), with good psychometric properties (Roberti, Harrington & Storch 2006). The 10 items are assessed on a scale of 1–5, with a total summed score up to 40. Lower scores (under 14) suggest low perceived stress, while higher scores (over 26) suggest high perceived stress. Previous research established Cronbach alpha within 0.74 and 0.91 (Lee 2012), while a Cronbach alpha coefficient of 0.90 was found in the present study. A high incidence of perceived stress was utilised to indicate lower well-being in the current study.

#### The Edinburgh Postnatal Depression Scale

The Edinburgh Postnatal Depression Scale (EPDS) is designed to assess for potential postnatal depression (Cox, Holden & Sagovsky 1987). It assesses positive and negative experiences in the past week and has good psychometric properties (Kernot et al. 2015). Scores are summed, and scores under 7 indicate minimal depression, where scores over 19 indicate severe depression. Scores over 7 and up to 13 indicate mild depression, while scores between 14 and 19 indicate moderate depression. The EPDS can be used in mothers with children outside of the postnatal period (Cox et al. 1996). Previous research established a Cronbach alpha coefficient of 0.87 (Cox et al. 1987). The present student yielded a Cronbach alpha coefficient of 0.83. As with perceived stress, the presence of depression was utilised to denote low well-being in the present study.

### Statistical analysis

Descriptive statistics were used to determine the incidence of maternal well-being. The pre- and post-test data were analysed using Statistical Package for the Social Sciences (SPSS) IBM, version 27, Chicago Illinois, USA. The Shapiro-Wilk test was used to calculate normality, and results confirmed the normal distribution of the data for the SWLS, SPANE, PSS-10 and EPDS, but not for the CSI-16 *(p* ≤ 0.001), therefore requiring non-parametric analysis for the CSI-16. To ascertain whether the incidence of life satisfaction and affect changed after the intervention paired samples t-tests were conducted, with *p* < 0.05 (2-tailed tests) regarded as indicative of statistical significance. To assess how BSI implementation influenced maternal couple satisfaction the Wilcoxon signed rank sum test for paired observations was utilised. Completed data sets at study entry were as follows: 114, 112, 103, 117 and 105 for the SWLS, SPANE, CSI-16, PSS-10 and EPDS, respectively. At 2-month follow-up completed data sets were as follows: 72, 70, 64, 75 and 68, for the SWLS, SPANE, CSI-16, PSS-10 and EPDS, respectively.

### Ethical considerations

The study was approved by the University of Pretoria Research Ethics Committee. The reference number is HUM051/0619. Participation was voluntary and informed consent was obtained. Confidentiality and anonymity were ensured during data collection and analysis. The authors observed the ethical standards required in terms of the University’s Code of Ethics for researchers and the Policy Guidelines for responsible research.

## Results

As presented in Table 1, the incidence of positive affect and satisfaction with life among the participants were relatively high before the intervention. Participants also reported moderate levels of satisfaction in their relationships; however, their range of scores was wide, as indicated by the high standard deviation in Table 1. Further, participants experienced moderate perceived stress and negative affect, with mild depression noted in the scores.

**TABLE 1:** The incidence of subjective well-being among mothers before the implementation of the sleep intervention.

Measure	*n*	M	SD	Observed range	Cronbach’s alpha coefficient
SWLS	114	27.68	4.88	11–35	0.85
SPANE-P	112	22.54	3.78	14–30	0.93
SPANE-N	112	15.37	4.40	6–26	0.87
CSI-16	103	63.77	11.85	25–80	0.96
PSS-10	117	18.76	7.48	2–35	0.90
EPDS	105	9.16	4.49	1–22	0.83

SWLS, satisfaction with life scale; SPANE-P, scale of positive experience; SPANE-N, scale of negative experience; CSI-16, couple satisfaction index; PSS, perceived stress scale; EPDS, Edinburgh postnatal depression scale; M, mean; SD, standard deviation.

The sample’s mean score on life satisfaction (*M* = 27.68) was moderate to high before the intervention. The mean scores on affect indicated a high presence of positive affect (*M* = 22.54) and moderate presence of negative affect (*M* = 15.37). Participants in this study had moderate couple satisfaction (*M* = 63.77) and perceived stress (*M* = 18.76). Finally, the participants’ mean EPDS score indicated mild depression for the sample (*M* = 9.16).

There were statistically significant differences in most facets of well-being 3 weeks after the conclusion of the intervention, as indicated in Table 2. Specifically, the mean scores for life satisfaction and positive affect were significantly higher in comparison to baseline, whereas scores for negative affect, perceived stress and postnatal depression were significantly lower. Levels of couple satisfaction did not change significantly. Further, increases in life satisfaction and positive affect were practically significant, with the Eta-squared statistic revealing medium (*η*^*2*^ = 0.10) and large (*η*^*2*^ = 0.17) effect sizes, respectively. The reduction in negative affect, perceived stress and depression was also practically significant, with the Eta-squared statistic revealing a large effect size (*η*^*2*^ = 0.32; *η*^*2*^ = 0.34 and *η*^*2*^ = 0.17) for negative affect, perceived stress and depression, respectively.

**TABLE 2:** Significance of differences in subjective well-being before and after the sleep intervention.

Measure	*n*	Pre-test		Post-test		*p*	*η* ^2^
		Mean	SD	Mean	SD		
SWLS	72	27.52	5.13	28.83	5.04	0.006**	0.10
SPANE P	70	22.70	3.76	24.24	4.12	0.000***	0.17
SPANE N	70	15.09	4.22	12.46	4.00	0.000***	0.35
CSI-16	64	62.80	12.49	63.94	12.47	0.133	-
PSS-10	75	18.12	7.89	13.59	6.79	0.000***	0.34
EPDS	68	8.34	4.48	6.63	4.18	0.000***	0.17

SWLS, satisfaction with life scale; SPANE-P, scale of positive experience; SPANE-N, scale of negative experience; CSI-16, couple satisfaction index; PSS, perceived stress scale; EPDS, Edinburgh postnatal depression scale; SD, standard deviation.

** , *p* < 0.01;

*** , *p* < 0.001.

## Discussion

The aim of this research was to determine the incidence of SWB for mothers reporting problematic child sleep and to explore changes in SWB following the implementation of a child sleep intervention programme at a sleep consultancy based in South Africa. At baseline, participants exhibited a high incidence of satisfaction with life and positive affect, but a mild incidence of depression, and moderate incidence of negative affect, relationship satisfaction and perceived stress. Although few studies have been conducted on parental satisfaction with life in the context of problematic child sleep, research indicates that, overall, parenthood is associated with higher life satisfaction (Baetschmann, Staub & Studer 2016). Further, prior research has determined that individuals generally have a high level of satisfaction with life (Diener et al. 2018), and as such, mothers of children with problematic sleep appear to be similar.

There is a paucity of research on positive affect and sleep. Prior research has revealed the mitigating impact of poor sleep on positive affect (Saksvik-Lehouillier et al. 2020). The high levels of positive affect in the present study, regardless of poor sleep, were therefore intriguing. This may be explained by prior research, which has revealed that parents within the first 6 months post-partum generally experience moderate to high positive affect (Giuntoli et al. 2020). Our findings therefore suggest that perceived child sleep problems do not appear to influence positive affect in mothers beyond that in other contexts of parenthood.

While research on parental negative affect when children present with sleep problems is limited, there is a wealth of literature focussing on poor sleep in different contexts, and prior research has determined that sleep deprivation heightens negative affect (Gordon & Chen 2014). Restricted sleep as a result of child sleep problems may therefore result in higher negative affect for such parents. The prevalence of negative affect in our sample aligns with extant literature on sleep deprivation in other settings. Our results therefore suggest that restricted sleep negatively impacts affect, irrespective of causation, and support should therefore be provided to ameliorate the effects of sleep deprivation and improve sleep, regardless of context.

The moderate levels of couple satisfaction reported in our study were unsurprising. Becoming parents frequently leads to more arguments and tension within a family (Kluwer 2010) and problematic child sleep is linked to higher stress in a relationship (Hiscock & Fisher 2015). Decreased social cognition, inhibition and empathy resulting from poor sleep may therefore heighten animosity and arguments (Gordon & Chen 2014). Nevertheless, the incidence of couple satisfaction in our sample indicates the experience of satisfactory relationships, despite problematic child sleep.

The participants’ moderate perceived stress is troubling. The findings align with that of prior studies associating problematic child sleep to heightened parental stress (Hall et al. 2017), indicating that despite cultural variation, mothers in Southern Africa experience similar stress as mothers in other countries. The observed incidence of stress may be because of challenging behaviour from tired children such as reluctance to go to bed and drowsiness during the day (Martin et al. 2019). Interventions to decrease maternal stress is therefore advised.

Problematic child sleep has been associated with depression in parents (Hall et al. 2017), and this relationship is both directly linked to maternal depression and reciprocal in that maternal depressive symptoms influence child sleep (Toffol et al. 2019). Therefore, the results of our study support that of previous research in other populations, associating problematic child sleep to elevation in depression for parents. This suggests that interventions for mothers of children with perceived sleep problems in Southern Africa may assist in decreasing postnatal depression (PND) in this population.

Although the mothers in this study experienced high life satisfaction and positive affect before the BSI, they also endured moderate couple satisfaction, moderate negative affect, moderate stress and mild symptoms of depression. The experience of negative affect, stress and depression, alongside moderate couple satisfaction, therefore suggests they had a moderate level of SWB before the intervention.

At follow-up, our results indicated improved levels of satisfaction with life, positive and negative affect, depression and stress although couple satisfaction remained unchanged. In this regard, sleep and life satisfaction are positively associated (Piper 2016). As a result of enhanced child sleep, mothers’ sleep may have improved in terms of both duration and quality, leading to improved life satisfaction. While further research is needed, this finding suggests that implementing BSI in this population may enhance satisfaction with life for mothers of children with problematic sleep despite the moderate effect size.

In reviewing the literature, there has been limited research investigating how BSI influences parental emotions. Existing research primarily focussed on negative affect, with results suggesting that negative affect decreases after BSI implementation (Hall et al. 2017; Symon & Crichton 2017). Our findings are therefore consistent with prior research and yield further support for BSI being effective in decreasing negative affect. Additionally, the elevation in positive affect aligns with Symon and Crichton’s (2017) observation that parents experienced a significant improvement in positive emotions following a BSI. However, they utilised a one-item scale to assess positive emotion (pleasure), while we measured a broader range of positive emotions, using the SPANE. It therefore enhances our knowledge of the potential impact of BSI on SWB in mothers, particularly in terms of positive affect.

Enhanced parental sleep after the BSI could potentially explain the amelioration in parental affect. Improved child sleep is likely to enhance the sleep of parents, and high sleep quality is associated with positive affect, while low sleep quality is associated with negative affect (Gordon & Chen 2014). Another possible explanation is that enhancements in satisfaction with life, depression and stress indirectly improved maternal affect following the BSI. Given the large effect size for both subscales, it is plausible that BSI implementation may contribute to improving affect in mothers of children with sleep problems, and our findings extend to mothers in Southern Africa.

Previous studies evaluating sleep interventions observed inconsistent results on whether couple satisfaction improves. Smart and Hiscock (2007) found no change in couple satisfaction following a BSI; however, three studies indicating improvement after BSI implementation were included in a review by Mindell et al. (2006). Additionally, facets such as religion, moral intelligence and differentiation of self, may influence couple satisfaction (Homaei 2019), independently of child sleep. Our results indicate that, while a tendency to improved couple satisfaction was noticed, it did not reach statistical significance. One potential reason is that although addressing problematic child sleep may enhance parental sleep quality and subsequently social cognition, inhibition and empathy, it may not necessarily resolve the conflicts associated with parenthood.

Studies have demonstrated that parental stress decreases following the introduction of sleep interventions (Field 2017). Our research showed a significant decrease in stress after implementing the BSI, and our results are therefore consistent with the literature associating sleep interventions and decreased stress. One possible reason for the decrease in reported stress could be attributed to the physical changes linked to improved parental sleep. Improved parental sleep is linked to increased child sleep following a sleep intervention (Hall et al. 2017). Furthermore, a concomitant result of BSI involves the enhancement of children’s daytime behaviour, such as increased alertness, improved mood and appetite and a sense of security in different settings (Hiscock et al. 2015). The stable schedules for daytime naps and bedtime included in sleep interventions may also assist in decreasing bedtime stress and physiological arousal (Mindell et al. 2015). These facets may therefore conceivably have led to decreased parental stress, and this hypothesis should be tested in future studies. These results provide additional support for the use of BSI in addressing parental stress when children present with sleep problems and may be useful to treat maternal stress in Southern Africa.

Prior research utilising GE or EwPP has continually reported notable reductions in maternal depression (e.g., Hall et al. 2017), which the findings of our study support. When neural activity is disrupted because of disturbed sleep, it regularises after obtaining sufficient sleep, therefore leading to improved mood as a result of reduced fatigue and improved sleep quality (Motomura et al. 2017). Setting limits at bedtime potentially contributes as well, in that it has been identified as a factor in the variation of parental depression scores (Hall et al. 2017). Sleep interventions involve educating parents on how to establish good child sleep habits and set boundaries around bedtime. Setting limits at bedtime therefore seems to lead to lower depression scores as a concomitant result. Lastly, the participants’ enhanced affect may have contributed to the decreased scores in depression although future studies should confirm this association. Behavioural sleep interventions may therefore potentially be utilised to assist mothers in sub-Saharan Africa who suffer from PND and who perceive their child’s sleep as problematic.

### Practical implications

The findings of our study support the association between motherhood and perceived life satisfaction and positive affect, suggesting that interventions targeting parental well-being broadly may focus on the positive emotional experiences associated with parenting.

The results revealing increased maternal negative affectivity when children present with sleep problems further emphasise the importance of addressing sleep problems to improve maternal well-being and indicate the requirement for targeted interventions focused on problematic child sleep as well as parental sleep quality. The study enhances our knowledge of the impact of sleep interventions on maternal SWB, particularly regarding affect, indicating the importance of considering a range of emotional experiences and outcomes when assessing the effectiveness of sleep interventions.

Despite the presence of child sleep problems, our findings indicate that mothers still experienced moderate levels of couple satisfaction. However, although there was a tendency for enhancement following the intervention, the outcomes did not show statistical significance, suggesting that factors beyond child sleep impact couple dynamics. Interventions aimed at improving parental well-being may therefore need to address broader aspects of relationship satisfaction independently from child sleep.

The stress and depression experienced by mothers who perceive their child sleep as problematic highlights the importance for interventions targeting stress and depression, both through improving child sleep and providing coping strategies for parents. Our results suggest potential value in sleep interventions incorporating changes to daytime and nighttime routines to reduce parental stress and enhanced sleep to reduce depression. Finally, the procedure used in our study incorporated intensive support from sleep consultants, potentially influencing the results and indicating a need for the development of more supportive behavioural sleep interventions in both clinical and community settings.

### Limitations

There are important limitations to our study. Firstly, analysis and findings are limited to mothers in committed relationships and results therefore cannot be generalised to fathers or individuals outside of committed relationships. Future studies should attempt to include fathers in data collection and broaden the inclusion criteria to parents outside of committed relationships.

Secondly, as there was no control group included in the study, it is not possible to ascertain whether the observed changes in SWB are attributable to sleep interventions specifically. Because the research was conducted in a community setting, parents could, upon request, change to a different intervention (either EwPP or GE). Comparison of the two intervention types is therefore not possible as some parents would have used a combination of both. Future studies should compare the specific influence of each intervention on the SWB of parents. Additionally, sampling was not random as parents selected to participate in the study, and the sense of agency experienced in addressing their child’s sleep problems might have influenced their well-being. The high attrition rate should also be explored through qualitative studies to determine barriers to implementing such interventions. Future studies should utilise randomised control trials to confirm the present findings and investigate alternative interventions to improve couple satisfaction.

Thirdly, the study procedure involved receiving thorough, daily assistance from sleep consultants, a practice that may not be common in all sleep intervention programmes. Findings from this study can therefore not be generalised to applications where sleep consultants engage in less support. Fourthly, the sample size of the current study was small, increasing the likelihood of a Type II error. Future studies should therefore include larger samples. Finally, given the timeframe of about 6–8 weeks between baseline and follow-up, normal maturation could conceivably have influenced scores on well-being. Longitudinal studies should be conducted to determine long-term impact on maternal well-being.

## Conclusion

Our results indicate that although mothers perceiving their child sleep as problematic in this Southern African sample reported high satisfaction with life and positive affect, thus indicating high levels of subjective well-being, the presence of moderate negative affect, couple satisfaction and perceived stress and mild incidence of depression indicate the potential for decreased subjective well-being. Our findings further suggest that implementation of sleep interventions, specifically graduated extinction and extinction with parental presence, may improve life satisfaction, affect, stress and depression, but not couple satisfaction, in the short term. This study contributes towards understanding the influence of restricted sleep on the subjective well-being of mothers and provides preliminary evidence regarding the benefits of sleep interventions for improving maternal subjective well-being in a Southern African sample.
